# Supplementary material for: Recurrent rectal foreign-body insertion in a child with caregiver-reported problematic short-form video use: A hypothesis-generating case report
Source: BMC Psychol. 2026 Sep 4;14:1399. doi: 10.1186/s40359-026-05507-0 (PMC13621643; doi:10.1186/s40359-026-05507-0)
Supplement: Supplementary file 1 [file 40359_2026_5507_MOESM2_ESM.pdf]

CARE case report guidelines

CARE Checklist of information to include when writing a case report

(cc) BY-NC-ND

Topic	Item	Checklist item description	Reported on Line
Title	1	The diagnosis or intervention of primary focus followed by the words "case report"	Line 1-2
Key Words	2	2 to 5 key words that identify diagnoses or interventions in this case report, including "case report".	Line 33-34
Abstract (no references)	3a	Introduction: What is unique about this case and what does it add to the scientific literature?	Line 15-31, 478-487
	3b	Main symptoms and/or important clinical findings	Line 55-66, 76-86, 158-181,Table 1
	3c	The main diagnoses, therapeutic interventions, and outcomes	Line 61-86, 93-128, 341-344， 371-378,Table 1
	3d	Conclusion—What is the main "take-away" lesson(s) from this case?	Line 28-31, 383-389， 469-476， 485-487
Introduction	4	One or two paragraphs summarizing why this case is unique (**may includereferences**)	Line 24-27,459-466
Patient Information	5a	De-identified patient specific information.	Line 52-54. Figure1, 2.
	5b	Primary concerns and symptoms of the patient.	Line 52-89, 162-169,Table 1
	5c	Medical, family, and psycho-social history including relevant genetic information	Line 52-54, 139-145
	5d	Relevant past interventions with outcomes	Line 113-128, 175-181,Table 1
Clinical Findings	6	Describe significant physical examination (PE) and important clinical findings.	Line.57-74, 82-91, 159-169,Table 1
Timeline	7	Historical and current information from this episode of care organized as a timeline	Figure 3
Diagnostic Assessment	8a	Diagnostic testing (such as PE, laboratory testing, imaging, surveys).	Line 57-74, 81-91, 236-250
	8b	Diagnostic challenges (such as access to testing, financial, or cultural)	Line 196-205,346-369,393-407
	8c	Diagnosis (including other diagnoses considered)	Line 294-303, Figure 1,Figure 2
	8d	Prognosis (such as staging in oncology) where applicable	Line 113-128,371-378
Therapeutic Intervention	9a	Types of therapeutic intervention (such as pharmacologic, surgical, preventive, self-care)	Line 93-119, Table 1, 340-344
	9b	Administration of therapeutic intervention (such as dosage, strength, duration)	Line 109,113-120,Table 1
	9c	Changes in therapeutic intervention (with rationale)	Figure 3,Table 1
Follow-up and Outcomes	10a	Clinician and patient-assessed outcomes (if available)	Line 119-128, Table 1,371-377
	10b	Important follow-up diagnostic and other test results	Table 1
	10c	Intervention adherence and tolerability (How was this assessed?)	Line 109-111,Figure 3,Table 1
	10d	Adverse and unanticipated events	Line 126-128,376-377
Discussion	11a	A scientific discussion of the strengths AND limitations associated with this case report	Line 346-369,383-407,431-476
	11b	Discussion of the relevant medical literature **with references**.	Line 196-205,207-315
	11c	The scientific rationale for any conclusions (including assessment of possible causes)	Line 208-315
	11d	The primary "take-away" lessons of this case report (without references) in a one paragraph conclusion	Line 24-31,297-299,478-487
Patient Perspective	12	The patient should share their perspective in one to two paragraphs on the treatment(s) they received	Line 409-429
Informed Consent	13	Did the patient give informed consent? Please provide if requested	Yes ☑ No ☐
