# Supplementary material for: Recurrent rectal foreign-body insertion in a child with caregiver-reported problematic short-form video use: A hypothesis-generating case report
Source: BMC Psychol. 2026 Sep 4;14:1399. doi: 10.1186/s40359-026-05507-0 (PMC13621643; doi:10.1186/s40359-026-05507-0)
Supplement: Supplementary file 2 [file 40359_2026_5507_MOESM1_ESM.docx]

**Author information**

| **Order of authors** | **Name** | **Degree** | **Gender** | **Email/** **ORCID** | **Organization and Address** |
| --- | --- | --- | --- | --- | --- |
| **First author** | Yunhe Wang¹ | Bachelor of Medicine | Male | [wangyunhe114@126.com](mailto:wangyunhe114@126.com)  https://orcid.org/0009-0000-5289-9503 | Pediatrics, Zibo Central Hospital, Shandong Provincial Regional Medical Center, No. 10 Shanghai Road, Zhangdian District, Zibo City, Shandong Province, China. |
| **Corresponding author** | Jie Zhao* | Doctor of Medicine | Female | [wyhsdzb@126.com](mailto:wyhsdzb@126.com)  https://orcid.org/0009-0007-2155-3658 | Pediatrics, Zibo Central Hospital, Shandong Provincial Regional Medical Center, No. 10 Shanghai Road, Zhangdian District, Zibo City, Shandong Province, China. |
| **Second author** | Xue Cheng² | Bachelor of Medicine | Female | zhuluoyu2012@163.com | Pediatrics, Zibo Central Hospital, Shandong Provincial Regional Medical Center, No. 10 Shanghai Road, Zhangdian District, Zibo City, Shandong Province, China. |
| **Second author** | Yuanyuan Xue² | Master of Medicine | Female | yuanyuan7501@126.com | Pediatrics, Zibo Central Hospital, Shandong Provincial Regional Medical Center, No. 10 Shanghai Road, Zhangdian District, Zibo City, Shandong Province, China. |
| **Second author** | Cong Diao² | Bachelor of Medicine | Female | 13561246571@163.com | Pediatrics, Zibo Central Hospital, Shandong Provincial Regional Medical Center, No. 10 Shanghai Road, Zhangdian District, Zibo City, Shandong Province, China. |
|  |  |  |  |  |  |
|  |  |  |  |  |  |
