# Supplementary material for: Recurrent rectal foreign-body insertion in a child with caregiver-reported problematic short-form video use: A hypothesis-generating case report
Source: BMC Psychol. 2026 Sep 4;14:1399. doi: 10.1186/s40359-026-05507-0 (PMC13621643; doi:10.1186/s40359-026-05507-0)
Supplement: Supplementary file 3 [file 40359_2026_5507_MOESM4_ESM.docx]

**Recurrent rectal foreign-body** **insertion in a child with caregiver-reported problematic short-form video use: A hypothesis-generating case report**

Yunhe Wang¹, Jie Zhao*, Xue Cheng², Yuanyuan Xue² and Cong Diao²

Corresponding author: Jie Zhao*, MD, Email: wyhsdzb@126.com

Pediatrics, Zibo Central Hospital, Shandong Provincial Regional Medical Center, No. 10 Shanghai Road, Zhangdian District, Zibo City, Shandong Province, China.

**Abstract**

**Background** Rectal foreign body refers to any object that enters the colon and rectum through the mouth or anus, becomes impacted, and cannot be expelled spontaneously. Foreign bodies in the pediatric gastrointestinal tract are relatively common in pediatric emergency departments; most of which are coins, fruit pits, button batteries, magnets, and toy parts ingested orally. Rectal foreign bodies in children are rare.

**Case Presentation** We report a rare case of a preschool child who experienced two episodes of rectal foreign body within one year. Through a retrospective analysis of the child’s behavior in daily life, we identified problematic short-form video usage, as clearly reported by his caregivers. By analysing the various possible mechanisms underlying the two instances of unusual behavior in this case, while also reviewing and analysing previous research on similar issues among adolescents, we conducted a further discussion and analysis of the relationship between the child’s unusual behavior and his excessive exposure to short-form videos, and put forward our own hypothesis. Finally, under the supervision and guidance of his parents, the child’s behavior has improved.

**Conclusion** We report a rare case of recurrent rectal foreign-body insertion in a preschool child. Our analysis leads us to hypothesise that there may be a potential link between the child’s unusual behavior and problematic use of short-form video apps. The putative association identified in this study is speculative and merely hypothesis‑generating; no definitive causal conclusions can be drawn. Larger‑scale studies are needed to validate or refute this potential association. In addition, when encountering similar preschool‑aged pediatric patients, clinicians should remind caregivers to pay attention to children’s media‑exposure status, and implement prudent supervision, patient guidance, and reasonable media use.

**Keywords** rectal foreign body, unusual behavior, problematic use of short-form video, excessive short-form video exposure, case report.

**Introduction**

Foreign bodies in the pediatric gastrointestinal tract constitute a medical emergency; delayed or improper management can lead to serious consequences and increase the burden on families and society. Rectal foreign bodies (RFB) are a type of gastrointestinal foreign body in children; though rare, they still pose significant risks. When a foreign body becomes impacted at a colonic or rectal bend or turn, symptoms of intestinal obstruction appear early. If left unchecked, this condition can progressively damage the mucosa, causing bleeding and ulcers. In severe instances, the bowel wall may perforate, leading to life-threatening emergencies such as bacterial peritonitis, intra-abdominal abscesses, septic shock, and major vessel rupture^1^. When this happens, children often require surgery. Unfortunately, these invasive and expensive procedures carry significant risks of postoperative complications, which can profoundly disrupt a child's physical growth and mental well-being. Studies in adults have shown that nearly 400 RFB removal surgeries are performed annually in the United Kingdom, with annual costs totaling 338,819 pounds, placing additional strain on the resources of the National Health Service^2^.

**Case presentation**

Patient X is a 4-year-old boy with no previous medical history and no family history of mental illness; his growth and development show no significant differences from those of healthy children of the same age and gender. On 27 January 2025, while playing at home, X inserted two markers into his anus. He subsequently experienced recurrent abdominal pain, was unable to bend over, and reported anal discomfort and involuntary fecal leakage; however, the markers had not been expelled. X informed his parents and was brought to our hospital’s A&E department three hours later. The A&E doctor performed a digital rectal examination and detected the lower end of the foreign body 5 cm from the anus; an attempt to remove it failed. An abdominal CT scan revealed a double-tubular low-density shadow approximately 15 cm in length within the descending and sigmoid colons, with no free gas visible in the abdominal cavity. Following completion of pre-operative investigations, we urgently performed a “colonic and rectal foreign body removal procedure” using a gastroscope (Olympus: GIF-HQ290, Japan). Two markers were located in the rectum, approximately 5 cm from the anus. Using an “electric snare (model: AG-5071-242523, Hangzhou AGS Medical Technology Co., Ltd., China)” to encircle the base of each marker and successfully removed them: both were black plastic markers approximately 15 cm in length; one had its cap intact, while the other was capless (the family later confirmed that the cap had been found at home and had not been inserted into the anus). (Figure 1)

**Figure 1: First incident of foreign bodies in the rectum.**

**A+B: abdominal CT images of X. B: shows a double-tubular, low-density shadow within the abdominal cavity (indicated by the long blue arrow). A: shows foreign bodies approximately 15 cm long.**

**C: two markers found in the rectum during colonoscopy. D: after removal of both markers.**

On 13 December 2025, while at home, X again inserted a pencil into his anus on his own. When he realized he couldn’t remove it and began to feel pain, he told his parents. An hour later, his parents brought him back to our hospital. His account was clear, and his parents had carefully verified before coming to the hospital that one of the two pencils they had just bought that afternoon was missing. X showed no obvious signs of intestinal perforation, so we did not perform an abdominal CT scan this time. An emergency “colonic and rectal foreign body removal procedure” was performed on him for a second time using a gastroscope (Olympus: GIF-HQ290, Japan). We observed the foreign body at the lower end of the rectum, approximately 5 cm from the anus. After encircling it with an “electric snare (model: AG-5071-242523, Hangzhou AGS Medical Technology Co., Ltd., China)”, we successfully extracted it. It was a green wooden pencil approximately 17 cm in length. Fortunately, neither end of the pencil had been sharpened, and no sharp lead was exposed. (Figure 2: E+D)

**Figure 2**: **Second incident of a foreign body in the rectum.**

**E: a pencil was found in the rectum during a colonoscopy. F: after removal of the pencil.**

**G+H: rectal mucosal injury due to a foreign body.**

Both of X’s incidents involving rectal foreign bodies were emergency cases, and we arranged for them to be prioritised via the emergency fast-track. Following an assessment, the anaesthetist reported that X had not been fasting for a sufficient period; therefore, general anaesthesia could not be administered until the required fasting period had been met. We explained in detail to X’s parents the respective advantages and disadvantages of pain-free endoscopy under anaesthesia compared to standard endoscopy while conscious, as well as the associated risks, potential complications and unforeseen events. They were very concerned about the risks associated with anaesthesia, while also wishing to minimise the duration of X’s discomfort as much as possible; ultimately, they opted for a standard endoscopy without general anaesthesia. To minimise X’s discomfort as much as possible, on both occasions we used the HQ290, which has a smaller diameter than a standard colonoscope (scope size: 10.2/9.9, i.e. maximum outer diameter of 10.2 mm); Prior to each procedure, 2% lidocaine hydrochloride gel was applied to the perianal area for local anaesthesia, while the scope itself was evenly coated with the gel to aid local anaesthesia and lubrication; the entire endoscopic procedure was performed gently and cautiously, with close observation of X’s signs of distress to adjust the force of manipulation and the volume of air insufflation, and brief rest periods were provided intermittently. Throughout the endoscopic procedure, X’s parents remained by his side to provide verbal reassurance. Both procedures proceeded smoothly and were successfully completed within 15 minutes. X’s vital signs remained stable throughout the entire process; he did not cry or display any other signs of non-cooperation, and no adverse events or unexpected incidents occurred.

During the first episode, endoscopy revealed only hyperaemia and oedema at the site where the foreign body was lodged in the colorectal mucosa; no obvious mucosal damage was observed. Following our assessment, we did not administer any medication. During the second incident, we observed multiple abrasions on his colorectal mucosa accompanied by slight oozing of blood. After repeated observations confirmed the absence of active bleeding, the procedure was concluded (Figure 2: G+H). Postoperatively, he was administered etamsylate injection (0.15 g, intravenous drip, twice daily) for haemostasis and other treatments. Following the removal of the foreign bodies on both occasions, X was observed for one day in the paediatric A&E observation ward. All vital signs remained normal and stable, with no signs of fever, abdominal pain, abdominal distension, bloody stools, vomiting or behavioral abnormalities. As the family had another child to care for at home, they voluntarily requested discharge the following day. The on-duty physician assessed that X showed no signs of secondary bleeding, infection or intestinal perforation, and, after providing detailed instructions on precautions to be taken, authorised their discharge for continued observation at home. During the two follow-up outpatient appointments, which took place three days after discharge on both occasions, and within one month of the operation, we received no reports or feedback from caregivers regarding the occurrence of the aforementioned symptoms or any other unexpected physical injuries.

Ethical approval for this case report was granted by our institutional review board (Approval No.: Research‑2025‑335). Written informed consent (drafted and signed in Simplified Chinese) for publication was obtained from the child’s legal guardian. Age‑appropriate assent of the child was considered during clinical management.

Prior to both endoscopic examinations, we conducted preliminary risk assessments in collaboration with the attending physician. We deliberately interviewed X’s parents separately, enquiring about the timeline and specific details of the two incidents, as well as X’s living environment, medical history, day-to-day behavior and conduct prior to the incidents; the responses we received were consistent and logical. They are an ordinary, middle-income family; relationships between the couple, between parents and children, and between siblings are harmonious. The interviews revealed no evidence of domestic violence, family trauma or other risk factors. X had no history of trauma leading to long-term organ damage; there were no accidental injuries prior to presentation, and no obvious symptoms of abdominal, urinary or perianal discomfort; there were no significant behavioral abnormalities prior to the first incident. Recently, he had exhibited episodes of repeated crying and tantrums following the suspension of the smartphone use (see below). While the parents were attending to administrative procedures, we deliberately avoided them and carefully questioned X in private: ‘How did the object get inside?’ The answer received was consistent: “I put it in myself.” In addition, we conducted a targeted physical examination of X: the skin over the entire body, particularly the genitalia, perianal region and oral mucosa, showed no obvious signs of erythema, oedema, scratch marks, ligature marks, lacerations, bleeding or ecchymoses; there was no sign of pain on palpation of muscles, bones or joints, nor any restriction of movement; nor were any old injuries, such as scars, found in the aforementioned areas. In both incidents involving foreign bodies, we identified no clear high-risk warning signs of child abuse; consequently, following the first incident, we limited our discussion to a verbal exchange between the endoscopy team and the attending physician, and did not organise a formal multidisciplinary consultation.

According to X's mother, X's father is rarely home due to work; she works from home to help support the family and takes care of him at the same time. In daily life, X inevitably cries and fusses for various reasons, which directly affects her work efficiency. Later, she discovered that watching short-form videos on her smartphone—the kinds X likes—was an effective way to reduce his crying and fussing; even watching videos for him when he was sick and needed to take medicine significantly improved his cooperation. X’s favorite video genres include cartoons, comedy, toys, and snacks. He watches an average of 1–2 hours per day, sometimes up to 4–5 hours. X is particularly fond of videos like “Ultraman Fighting Monsters”; while watching, he’ll dance around and mimic the characters. However, she avoids playing these types of videos for X before bedtime, because she’s noticed that watching them makes it impossible for him to stop. He’d rather stay up and watch one after another than go to sleep, and it’s very difficult to stop him, which also leaves him lethargic the next day. Sometimes, even without watching such videos before bed, X will perform the signature “Ultraman vs. Monsters” moves in his sleep.

Around mid-November 2025, X’s mother happened to notice that he was laughing heartily after watching a prank video—in which someone poked another person on the bottom—that had been recommended to him by a short-form video app. Coincidentally, she herself had also inadvertently received similar videos pushed by the app while using it. This raised red flags for her. Although she wasn’t sure at the time whether these videos were related to X’s unusual behavior, she decided to try restricting his phone use. X reacted very aggressively; his endless demands for the phone and repeated tantrums caused her great distress. She tried various solutions: replacing the app with TV shows, playing games with him, and buying toys or snacks to distract him. However, on the odd occasion, she would compromise; at other times, she would simply remain silent and give him the cold shoulder. In addition, she discovered that X had, on two occasions, secretly taken her smartphone while she was asleep to watch videos. Even so, she had not anticipated that X would engage in self-insertion again.

Following the second incident, the clinical risk level rose significantly. On 14 December 2025, while X was under observation in the paediatric accident and emergency ward, we (paediatric gastroenterologists), in collaboration with the initial consulting physicians, senior paediatricians, paediatric health care specialists, nursing staff involved in endoscopic procedures, and a surgeon, initiated a formal multidisciplinary team (MDT) discussion and a child protection assessment. We conducted a comprehensive review of the full medical history, interviews with the family and the child, and repeated targeted physical examinations. Child maltreatment was considered during the evaluation. Based on the available clinical assessment, separate caregiver interviews, developmental evaluation (see below), and multidisciplinary review, no evidence substantiating abuse was identified. Finally, we concluded that this case did not require formal referral to external social work agencies or statutory child protection services.

**Methods**

We previously attempted to quantify and assess X’s behavior using tools such as the Adolescent Pathological Internet Use Scale (APIUS)^3^, the Digital Addiction Scale for Children (DASC)^4^, the Video Game Addiction Questionnaire (VGAQ)^5^, and the Social Media Addiction Questionnaire (SMAQ)^6^, but found that these scales were not suitable for children in this age group. This is because they are too young to describe their own feelings, and evidence of unusual behavior comes primarily from caregivers’ observations and subjective assessments. It should be noted that there are currently no internationally validated diagnostic criteria for short-form video addiction or media addiction applicable to preschool children. Therefore, the term “problematic short-form video usage” used in this paper serves solely as a descriptive term for behavioral patterns explicitly reported by caregivers and does not constitute a formal clinical diagnosis.

**Discussion**

Several studies have noted that child abuse is a common cause of RFB in children^7,8^. As mentioned earlier, we found no evidence of abuse in this child, and the stark contrast between X’s emotional responses and those of the parents during the two colonoscopies went some way towards allaying our concerns in this regard. During both endoscopic procedures, despite taking a number of measures to help X minimise discomfort as much as possible, we were unable to achieve the level of comfort experienced under general anaesthesia. X’s parents were devastated by the sudden blow, calling it “unbelievable and impossible to accept!” However, X showed no fear or resistance throughout the procedure; his cooperation during treatment astonished us. After we removed the foreign bodies, X even thoughtfully comforted his mother: “Mom, I was brave—I didn’t cry!”

The repeated insertion of foreign objects into the rectum by preschool children may be mediated by a variety of mechanisms. During the MDT meeting on 14 December 2025, we discussed and analysed X’s repeated self-insertion behavior and its underlying psychological and behavioral mechanisms. Preschoolers' impulse control skills are not yet fully developed; inadequate impulse control regulation may be a contributing factor in this case. Furthermore, following the parents’ initial intervention, impulsive behaviors such as crying and tantrums increased significantly; however, based on our analysis, this does not seem to readily explain the high degree of similarity between his two episodes of unusual behavior. The seeking of sensory stimulation is another differential diagnosis that needs to be considered; however, the caregivers’ reports from daily observations revealed no clear behaviors in X suggestive of sensory‑seeking stimulation.

In clinical practice, behavioral reinforcement within the family is a common cause of repetitive behavior. As X’s mother actively implemented a series of behavioral management strategies upon discovering the prank video, it is unlikely that this mechanism was the primary driving factor. A simple relapse following the initial episode is also a plausible possibility; however, this does not appear to account for the temporal association between the increase in X’s exposure to relevant short-form video content and the recurrence of the behavior prior to the second incident.

14 December 2025, before the MDT discussion, specialists from our hospital’s Child Health Department administered the “Achenbach Child Behavior Checklist^9^” (CBCL, CBCL – Chinese Version for ages 4–16^10^) to the parents to screen for factors such as X’s social withdrawal, depression, immaturity, somatic complaints, sexual concerns, schizotypal behavior, aggression and disciplinary issues. The assessment results indicated no significant emotional or behavioral risks. They assessed X using the “Developmental and Behavioral Assessment Scale for Children Aged 0–6 (WS/T 580—2017)^11^”, and the results indicated that X’s overall developmental quotient was at a moderate level (gross motor skills: good; fine motor skills, adaptive skills, language and social behavior: all moderate). They again invited X’s mother to complete the “The Swanson, Nolan, and Pelham scale version IV (SNAP-IV^12^, SNAP-IV – Chinese Version for Parents and Teachers^13,14^)”, the results of which showed that X’s performance was normal across the dimensions of attention deficit, hyperactivity and impulsivity, and oppositional defiant behavior. As X did not exhibit any obvious tic disorders, autism or other significant abnormalities, they did not conduct any further in-depth or extended investigations. The final assessment conclusion was: no features were identified that clearly indicated a potential neurodevelopmental disorder. It should be noted that, as our hospital does not have a specialist paediatric psychiatry or psychology department, no full-time paediatric psychiatrist or psychologist was involved in the face-to-face interviews or the MDT assessment; consequently, there was a lack of structured assessment and diagnosis by specialists in paediatric psychiatry or psychology. Furthermore, the assessment scales mentioned above are based on the carer’s observations of X’s past behavior; no questionnaire-based screening was conducted with the teachers at X’s nursery school, and there may therefore be a certain degree of self-report bias. All of these factors may contribute to limitations in the assessment.

After the second colonoscopy, we asked X carefully, “Why did you do that?” He replied, “I thought it was fun, and I wanted to try it.” Developmental exploratory behavior is another important factor we have considered. Studies have shown that during the early stages of sexual exploration, boys are more likely to engage in self-exploration of the anus and rectum^15,16^. Having had an unpleasant experience during a previous endoscopy, we are left wondering whether X’s act of inserting an object into the same body cavity again constitutes exploratory behavior. Research suggests that four-year-olds tend to choose activities that are more novel and offer greater opportunities for learning^17^.

The impact of innovations brought about by the digital age on our lives is evident. Social media platforms on smartphones are a double-edged sword: while they provide convenience and entertainment for our work, study, and daily lives, they also have drawbacks, including user addiction, cognitive biases in some shared content, and widely varying video quality—sometimes even containing inaccurate information^18,19^. Several studies have shown that these social media platforms use exceptionally powerful content recommendation algorithms. These algorithms analyze users’ personal preferences based on data such as time spent on the platform, likes, comments, and shares, precisely matching similar content that users might find interesting and prioritizing it for higher visibility^18,20^. Consequently, we cannot help but be concerned about the repeated exposure of certain videos containing inappropriate information to preschool children, a particularly vulnerable demographic.

Strong curiosity, poor risk judgment, inadequate self-protection awareness, and a propensity for naive imitation and exploratory behavior characterise preschool children in this age group. In daily life, whether it be the various subtle hints hidden in short-form videos on mobile phones or the provocative scenes in television dramas, these may unconsciously influence their psychological and behavioral development, and there is a possibility that they may exhibit atypical imitative behavior.

A study by Caroline Brand et al. found that adolescents exhibiting addictive social media behavior have a 19.0% to 25.2% probability of developing psychosocial health problems^21^. Gadi Lissak’s research indicates that addictive behavior related to digital media can cause structural changes in brain regions associated with cognitive control and emotional regulation^22^. Numerous studies have also examined the misleading influence and adverse effects of digital media platforms, such as TikTok, on the physical and mental health of children and adolescents^20,22–24^. Admittedly, social media platforms have set minimum age requirements for registration and generally offer a “Minor Mode,” however, the reality is that there are times when children are watching videos on the platform using their caregivers’ phones. This situation should likewise prompt our vigilance and attention.

As reported by caregivers: the child exhibited excessive short‑form video exposure and difficult‑to‑limit screen‑time use, with a possible history of exposure to prank‑type content. Improvement was reported following adjustments to supervision and smartphone access (see Follow‑up section for details). After analyzing these factors, we generated a hypothesis that a potential association might exist between the child’s problematic short‑form video use and his unusual behavioral presentations. Nevertheless, we cannot establish causality between the two. After all, alternative explanations remain possible, such as developmental curiosity, developmental exploratory behavior, imitative behavior, impulsivity, sensory‑seeking behavior, family behavioral reinforcement, simple recurrence after the first episode, environmental access to objects, and supervision‑related factors. Furthermore, these aforementioned factors may coexist and are not mutually exclusive.

In its article “Global action on problematic usage of the internet,” The Lancet calls for more action to protect individuals—especially children and adolescents—from the potential adverse effects of social media use^25^. Research has confirmed that three behaviors—early exposure to digital devices, using electronic devices before bedtime, and using them alone—all have lasting adverse effects on children^26^. After consulting with experts from our hospital’s Pediatric Health Care Department, we (pediatric gastroenterologists in collaboration with senior pediatricians) advised them to watch videos with their child while managing screen time. This approach not only helps them understand why their child enjoys such videos but also promotes parent-child interaction. At the same time, it enables early detection of issues that may arise during the child’s development, helping to redirect their attention so they can be properly guided and corrected. Research by DeLago C et al. also supports this view: caregivers’ monitoring of children’s media use and supervision during play helps reduce the likelihood that children will engage in or contemplate inappropriate behavior^8^.

**Follow-up:**

**Figure 3**: **Timeline.**

| **Table 1. Longitudinal Follow‑up: Pediatric Problematic Smartphone Use with Rectal Foreign Body Case** | | | | | | |
| --- | --- | --- | --- | --- | --- | --- |
|  |  |  |  |  |  |  |
| **Date** | **Maximum daily smartphone requests (times/day)** | **Screen time (hours/day)** | **Key behavioral observations** | **Intervention response summary** | **Unauthorized phone taking** | **Recurrence of self-insertion** |
| 12/17/25 | Up to 3–4 | ~2–3 | Docile and timid; fixated on preferred short videos; coaxing requests. | Supervised short videos accepted; no other interventions assessed at baseline. | None | No recurrence |
| 12/24/25 | ~7–8 | Up to 3–4 (difficult to control) | Swiped past educational or life-skill videos without hesitation. | Toys/snacks most effective; play effective; TV and verbal praise ineffective for diversion. | Once (mother’s phone during cooking) | No recurrence |
| 12/31/25 | ~6–7 | Difficult to control (similar to prior) | Preferred personal-interest short videos; uninterested in educational content. | Toys/snacks most effective; play effective; TV and verbal praise ineffective. | None | No recurrence |
| 01/07/26 | ~6–7 (similar to prior) | Slightly shorter; estimate unclear | Watched educational videos under supervision, but with poor concentration. | Toys/snacks most effective; play effective; verbal praise somewhat effective but often ineffective. | Once ( mother’s phone while receiving guests) | No recurrence |
| 01/14/26 | ~5–6 | Up to ~3 (difficult to control) | Watched educational videos longer than before; actively chose TV over them. | Toys/snacks most effective; play effective; verbal praise effectiveness noticeably improved. | None | No recurrence |
| 01/24/26 | ~5–6 (similar to prior) | Slightly shorter than prior | Watched educational videos longer; more willing to watch TV than educational videos. | Toys/snacks most effective; play effective; verbal praise effective but not on every occasion. | None | No recurrence |
| 02/04/26 | ~4–5 | ~2, up to 3 | Asked questions when unable to understand educational videos; more willing to watch TV. | Toys/snacks most effective; play effective; verbal praise effective most of the time. | Once (father’s phone while sleeping) | No recurrence |
| 02/14/26 | ~4–5 | ~2 | Asked more questions than before; enjoyed recognizing animals. | Toys/snacks most effective; play effective; verbal praise effective most of the time. | None | No recurrence |
| 02/28/26 | 3–4 | Slightly shorter; estimate unclear | Asked the most questions about educational videos on general life knowledge. | Toys/snacks most effective; play effective; verbal praise effective, occasionally ineffective. | None | No recurrence |
| 03/14/26 | ~3–4 (similar to prior) | ≤2 (longest ~2) | Sometimes imitated short video content to learn life skills (dressing, bathing). | Toys/snacks most effective; play effective; verbal praise effective, occasionally ineffective. | None | No recurrence |
| 03/29/26 | 2–3 | Slightly shorter; estimate unclear | Increased engagement in life-skill learning (sharpening pencils, peeling apples). | Toys/snacks most effective; play effective; verbal praise effective. | None | No recurrence |
| 04/13/26 | 1–2 | 1–2, rarely >2 | Increased life-skill learning behaviors (e.g., organizing toys). | Toys/snacks most effective; play effective; verbal praise effective. | None | No recurrence |
| 04/29/26 | 1–2 | Slightly shorter; estimate unclear | Was learning abstract concepts (days of week, time of day) with frequent errors. | Toys/snacks most effective; play effective; verbal praise effective. | None | No recurrence |
| 05/14/26 | NA (phone unanswered) | NA (phone unanswered) | NA (phone unanswered) | NA (phone unanswered) | None | No recurrence |
| 05/24/26 | NA (phone unanswered) | NA (phone unanswered) | NA (phone unanswered) | NA (phone unanswered) | None | No recurrence |
| 06/01/26 | 1–2 (May); now occasional | 2–3 during family illness; decreased after | Could state days of week and time of day; recited numbers; reverse order very difficult. | Toys/snacks most effective; play and verbal praise effective. | None | No recurrence |
| 06/13/26 | Occasional | 0.5–1, sometimes 1–2 | Was learning to recite Tang poetry, but frequently made errors. | Toys/snacks most effective; play and verbal praise effective. | None | No recurrence |
| 06/20/26 | Occasional | Mostly 0.5–1, occasionally >1 | Could recite 2 Tang poems. | Toys/snacks most effective; play and verbal praise effective. | None | No recurrence |
|  |  |  |  |  |  |  |
| Note. NA = not available. | | | | | | |
| Data were obtained via caregiver-reported telephone follow-up. | | | | | | |
| Screen time and smartphone request frequency represent caregiver-estimated maximum daily values during the interval preceding each follow-up. | | | | | | |
| Visit 1(12/17/25) represents the baseline outpatient assessment. | | | | | | |

We conducted a six-month telephone follow-ups on X’s subsequent behavioral presentations (see Table 1. Longitudinal Follow up: Pediatric Problematic Smartphone Use with Rectal Foreign Body Case). Detailed follow-up information is provided in Supplementary File 1, titled “*Longitudinal follow-up summary of behavioral interventions in a pediatric case of problematic smartphone use and rectal foreign body (December 2025 – June 2026)*”. Following the outpatient visit for the second rectal foreign‑body event, general pediatricians and pediatric gastroenterologists on our team pre‑analyzed and discussed follow‑up procedures and details to develop a follow‑up protocol, which originally planned weekly telephone follow‑up assessments. In practice, however, the scheduled follow‑up plan proved difficult to implement, and caregivers expressed concerns regarding disruption to their daily lives. Ultimately, we flexibly arranged subsequent follow‑up timelines (at least twice per month) according to X’s family circumstances and behavioral presentations reported by the caregivers. In addition, two follow‑up attempts on May 14 and May 24, 2026 went unanswered. We later learned that X’s grandfather had been hospitalized for illness, leaving the family unavailable to take or return our calls.

During follow‑up assessments, we prioritized documentation of the following metrics: maximum daily smartphone requests, screen‑time duration, performance under supervised short‑form‑video viewing, substitution effects of television viewing, new toys and snacks, play and games, and verbal praise or incentives. We also documented whether “unauthorized smartphone access”, “recurrent self‑insertion behavior”, or “other unusual behavioral presentations or adverse events” occurred. Based on caregiver feedback, we offered recommendations for subsequent interventions, including patient guidance, increased companionship, enhanced verbal encouragement, supervision of short‑form‑video viewing, reduced opportunities for unauthorized access to smartphones, development of new interests, and gradual life‑skills‑training implementation.

From feedback reports provided by caregivers, we experienced substantial difficulty quantifying and evaluating changes in X’s behavioral presentations using concrete numerical items. During follow‑up assessments, we attempted to collect and document the following metrics: average daily frequency of each intervention factor, duration and proportion of smartphone versus television viewing, duration and proportion of educational or life‑skill‑enhancing video viewing, duration of play or games, duration of caregiver companionship, daily duration of life‑skills training, exact sleep duration, and the frequency and timing of restless behaviors during sleep. These collection efforts were unsuccessful. The primary reasons were that such information was so difficult to obtain, and caregivers provided insufficiently detailed and precise descriptions of X’s behavioral details. Most of their responses contained vague qualifiers such as “approximately”, “probably about the same”, and “similar to the last time”. Excessive probing would increase their concerns about disruptions to their daily lives. For instance, during the follow‑up on April 29, 2026, X’s mother stated: *During telephone follow‑up, the child would listen silently nearby, and she was concerned that this might adversely affect the child.*

Furthermore, we also encountered numerous difficulties and challenges in obtaining data for the two items “Maximum daily smartphone requests” and “Screen time” within the follow‑up form. For example, during the telephone follow‑up in the first week, we received the following feedback from the family: “On the day we went to the amusement park, X actively asked for the smartphone only 3‑4 times within one day, with screen‑time lasting merely 1‑2 hours. However, these figures doubled on the day after returning home.” Consequently, we had to record the maximum achievable frequency or maximum duration observed within a single day during a follow‑up period. Accordingly, we only documented these data as objectively reported by caregivers and cannot guarantee that some of these data truly reflect X’s behavioral status or changes. It should be emphasized that caregivers’ descriptions for these two variables were also often vague.

As of June 20, 2026, X occasionally asked for the smartphone; however, caregivers observed no obvious crying or tantrums associated with these requests, and caregiver‑reported screen‑time duration was reduced compared with prior assessments. With caregivers’ accompaniment and supervision, X engaged in moderate short‑form‑video viewing, primarily using short‑form videos to acquire knowledge and life‑skill‑related content. There had been no episodes of unauthorized smartphone access for four consecutive months. No recurrent self‑insertion events occurred over more than six months, and no new unusual behavioral presentations or other adverse events were noted. During the June 20, 2026 follow‑up contact, caregivers stated that X was currently in very good condition and that they did not wish their daily lives to be overly disturbed. Accordingly, we terminated follow‑up, and longer‑term recording, analysis, or verification of subsequent changes or improvements in X’s behavioral presentations could not be performed.

Following the first colonic foreign‑body incident, we did not conduct standardized telephone follow‑up. We only provided our contact information to the caregiver during the outpatient follow‑up visit on January 31, 2025, and instructed the caregiver to contact our team promptly or return for clinical re‑evaluation should postoperative complications or other concerns arise. In hindsight, we recognize that standardized follow‑up after the first incident might have enabled us to identify excessive screen‑time exposure in X at an earlier stage, allowing us to implement additional interventions to prevent the second incident.

We have documented all information regarding X’s behavioral manifestations collected from clinical procedures and MDT interviews following the second incident in the present manuscript. Nevertheless, many records throughout the clinical course are incomplete and lack sufficient granularity. Examples include the full duration of X’s excessive screen‑time exposure (e.g., months or years) and its specific content. Of particular note are the exact timestamps and details of key events occurring between the two incidents: the date when X laughed happily after watching prank‑themed videos; how many such videos the caregiver observed X viewing in daily life; the specific dates and frequency at which X’s mother received similar short‑video push notifications again; the exact time when the caregiver decided to restrict X’s smartphone access; X’s screen‑use duration and more granular behavioral details prior to the second incident; as well as the specifics, implementation status, and effectiveness of all interventions already undertaken by the caregiver at that time. These limitations arose because the caregiver could only provide vague descriptions at the time, such as “I cannot recall the exact dates/details” or “It seemed to be approximately…”. Frankly, we did not recognize the significance of these data at that point; this was our first encounter with such a case, and no prior experience was available for reference. Given that follow‑up has been terminated, the aforementioned information can no longer be retrieved or supplemented. This incompleteness of data constrains our analyses and clinical interpretations.

**Parents perspective**

His parents describe X as a “lively and smart” boy who has shown a strong sense of independence and a willingness to take risks from a very young age. One day, while playing soccer with friends his age, the ball rolled into a roadside ditch. The other children were afraid to go down because it was too deep, but he was the only one brave enough to jump in and retrieve it. When he got home, his mother asked how he had managed to get out. He explained that before jumping in, he had found a long, thick, dry branch by the roadside. Together, they pushed the branch down along the edge of the ditch to form a makeshift ladder. After retrieving the ball, he climbed back up using the branch as a step.

X has an older brother who attends junior high school. As the youngest child, X has historically received more attention than his sibling. Due to their busy work schedules, they did not have enough time to spend with their children. At first, they thought that X watching short-form videos on their smartphones was simply a form of innocent play, much like playing games, and they did not feel any cause for concern, as X’s day-to-day behavior was no different from that of other children. It was only after these two incidents involving foreign objects in the rectum that they realised X was exhibiting unusual behavior. They believe the first instance of unusual behavior could be explained as a coincidence, but the second is much harder to dismiss as such. They made it clear that: the problematic use of short-form videos apps is a significant contributing factor to X’s unusual behavior, as they have been unable to find any other evidence in X’s daily life that is more relevant or convincing. They hope these two incidents will not leave X with psychological trauma or lingering effects, and that X will continue to grow up healthy, both physically and mentally.

**Limitations and Significance of the Study**

This is a single‑case study entirely reliant on retrospective reports from caregivers. There is no objective verification of full‑course exposure regarding the video‑use behaviors reported by caregivers, leading to incomplete data across the entire clinical course. We only arranged telephone follow‑up after the second rectal foreign‑body incident, with no regular in‑person outpatient visits or home‑based interviews. No dedicated child psychologist or child psychiatrist participated in the MDT assessment or subsequent follow‑up procedures. Furthermore, all interventions in this study were non‑standardized and primarily led and implemented by family members.

All information concerning the full trajectory of X’s behavioral changes and subsequent improvements was derived exclusively from caregivers’ verbal accounts, which may introduce recall bias and confounding factors. Many descriptions provided by caregivers lacked sufficient detail, precision, and completeness. Some data collected via telephone follow‑up may not truly reflect X’s behaviors or behavioral changes. In addition, no validated standardized behavioral or media‑use assessment tools were applied to measure X’s behavioral changes or improvements throughout the process. Moreover, long‑term follow‑up was limited in duration, and follow‑up attempts for certain time periods were unsuccessful.

Collectively, these limitations indicate that this study cannot establish that problematic short‑video use caused the child’s recurrent rectal foreign‑body insertion behaviors. The observed behavioral improvements represent only caregiver‑reported phenomena and cannot be directly attributed to the implementation of supervision strategies and mobile‑phone‑use interventions. Accordingly, findings from this case can only generate research hypotheses and are not generalizable to broader populations. Future studies with larger sample sizes, validated objective assessment tools, full‑cycle multidisciplinary‑team involvement, and detailed prospective documentation and tracking are warranted to further explore the potential association between problematic short‑video use and unusual physical behaviors among preschool‑aged children.

Current research of this kind has primarily focused on adolescents, with few studies involving preschool children. Although there are some similarities between these two groups, the conclusions drawn from the studies on adolescents that we have cited cannot be directly extrapolated to this case. The value of this report lies in its observation and documentation of the clinical presentation and diagnostic clues in this case involving a preschool child. It provides a new perspective on the physical and behavioral abnormalities, as well as the mental and physical harm associated with problematic use of new media, in this age group, and lays the foundation for subsequent basic research and cohort studies involving this population. We also hope that our hypotheses can be either validated or falsified by large-sample, multi-centre data.

Furthermore, we only initiated a formal multidisciplinary child protection assessment following the second inciden. Although no obvious signs of abuse were identified during the initial screening at the first consultation, this case nevertheless serves as an important reminder that clinical vigilance must be maintained even after a single incident of rectal foreign body insertion in a preschool child. The absence of obvious suspicious signs at the initial consultation does not entirely rule out the risk of subsequent recurrence; had a multidisciplinary child protection consultation been conducted at the first visit, behavioral interventions might have been implemented at an earlier stage to avoid repeated episodes.

**Conclusion**

We report a rare case of recurrent rectal foreign-body insertion in a preschool child. Our analysis leads us to hypothesise that there may be an potential link between the child’s unusual behavioral manifestations and problematic use of short-form video apps. It is important to emphasize that the putative association observed in this case is speculative and intended solely to generate research hypotheses. Given the multiple limitations of this study, definitive causal conclusions cannot be drawn from the available data. Larger‑scale studies are expected to either validate or falsify this potential association. In addition, when encountering similar preschool‑aged pediatric patients, clinicians should remind caregivers to pay attention to children’s media‑exposure status, and implement prudent supervision, patient guidance, and reasonable media use.

**Abbreviations**

RFB: Rectal Foreign Body

MDT: Multidisciplinary Team

APIUS: Adolescent Pathological Internet Use Scale

DASC: Digital Addiction Scale for Children

VGAQ: Video Game Addiction Questionnaire

SMAQ: Social Media Addiction Questionnaire

CBCL: Achenbach Child Behavior Checklist

SNAP-IV: The Swanson, Nolan, and Pelham scale version IV

**Acknowledgments**

The authors acknowledge and thank the patients for consenting to share their experiences.

**Author contributions**

YW: Investigation, Conceptualization, Data curation, Methodology, Writing – original draft, and Writing – review & editing. JZ: Conceptualization, Supervision, Validation, Writing – original draft, and Writing – review & editing. XC, YX, and CD: Writing – original draft and Writing – review & editing. All authors read and approved the final manuscript.

**Funding**

The authors did not receive any specific grant from funding agencies in the public, commercial, or nonprofit sectors for this work.

**Data availability**

The original contributions presented in the study are included in the article. Further inquiries can be directed to the corresponding author(s).

**Declarations**

**Ethics approval and consent to participate**

This study was conducted in accordance with the Declaration of Helsinki and its subsequent amendments. The Ethics Committee of the Zibo Central Hospital of Shandong Province approved the study (Approval No.: Research‑2025‑335). The patient's parents provided written informed consent (drafted and signed in Simplified Chinese) for participation in this study.

**Consent for publication**

Written informed consent (drafted and signed in Simplified Chinese) was obtained from the parents for the publication of any potentially identifiable images or data included in this article.

**Competing interests**

The authors declare no competing interests.

**Publisher’s Note**

Springer Nature remains neutral on jurisdictional claims in published maps and institutional affiliations.

**References**

1. Odagiri H, Yasunaga H, Matsui H, Fushimi K, Iizuka T, Kaise M. Difference in Outcomes of Rectal Foreign Bodies between Males and Females: A Retrospective Analysis of a National Inpatient Database in Japan. Digestion. 2015;92(3):165-70. doi: 10.1159/000439125. Epub 2015 Sep 10. PMID: 26352207.
2. Bhasin S, Williams JG. Rectal foreign body removal: increasing incidence and cost to the NHS. Ann R Coll Surg Engl. 2021 Nov;103(10):734-737. doi: 10.1308/rcsann.2020.7129. PMID: 34719960; PMCID: PMC10335310.
3. Lei L, Yang Y. The development and validation of the Adolescent Pathological Internet Use Scale (APIUS) [article in Chinese]. Acta Psychol Sin. 2007;39(4):688-696.
4. Hawi NS, Samaha M, Griffiths MD. The Digital Addiction Scale for Children: Development and Validation. Cyberpsychol Behav Soc Netw. 2019 Dec;22(12):771-778. doi: 10.1089/cyber.2019.0132. Epub 2019 Nov 22. PMID: 31755742.
5. Bagot KS, Tomko RL, Marshall AT, Hermann J, Cummins K, Ksinan A, Kakalis M, Breslin F, Lisdahl KM, Mason M, Redhead JN, Squeglia LM, Thompson WK, Wade T, Tapert SF, Fuemmeler BF, Baker FC. Youth screen use in the ABCD® study. Dev Cogn Neurosci. 2022 Oct;57:101150. doi: 10.1016/j.dcn.2022.101150. Epub 2022 Sep 1. PMID: 36084446; PMCID: PMC9465320.
6. Hawi NS, Samaha M. The Relations Among Social Media Addiction, Self-Esteem, and Life Satisfaction in University Students. Social Science Computer Review. 2017 Oct;35(5):576–86. doi:10.1177/0894439316660340.
7. Ayantunde AA. Approach to the diagnosis and management of retained rectal foreign bodies: clinical update. Tech Coloproctol. 2013 Feb;17(1):13-20. doi: 10.1007/s10151-012-0899-1. Epub 2012 Sep 20. PMID: 22993140.
8. DeLago C, Schroeder CM, Cooper B, Deblinger E, Dudek E, Yu R, Finkel MA. Children who engaged in interpersonal problematic sexual behaviors. Child Abuse Negl. 2020 Jul;105:104260. doi: 10.1016/j.chiabu.2019.104260. Epub 2019 Nov 25. PMID: 31776010.
9. Achenbach TM, Edelbrock CS. Behavioral problems and competencies reported by parents of normal and disturbed children aged four through sixteen. Monogr Soc Res Child Dev. 1981;46(1):1-82. PMID: 7242540.
10. Ren’e X, Huiqin T, Zhixiong Z, Zhiping C. A survey on behavioral problems of 24,013 school children from 26 units of 22 provinces in China— survey on mental health problems among “only-child”, prevention and standardization of Achenbach Child Behavior Checklist in China. Shanghai Arch Psychiatry. 1992;4(1):47–55.
11. National Health Commission of the People’s Republic of China. Developmental and Behavioural Assessment Scale for Children Aged 0–6 (WS/T 580—2017) [Internet]. Beijing: National Health Commission of the People’s Republic of China; 2017 Oct 12 [cited 2026 Aug 09]. Available from: https://www.nhc.gov.cn/wjw/c100311/201710/998011ad285a45808bfff259da50c125.shtml.
12. Swanson JM. School-Based Assessment and Interventions for ADD Students: Swanson, Nolan, and Pelham Scale Version IV (SNAP-IV). Irvine (CA): KC Publications; 1992.
13. Bussing R, Fernandez M, Harwood M, Wei Hou, Garvan CW, Eyberg SM, Swanson JM. Parent and teacher SNAP-IV ratings of attention deficit hyperactivity disorder symptoms: psychometric properties and normative ratings from a school district sample. Assessment. 2008 Sep;15(3):317-28. doi: 10.1177/1073191107313888. Epub 2008 Feb 29. PMID: 18310593; PMCID: PMC3623293.
14. Gau SS, Shang CY, Liu SK, Lin CH, Swanson JM, Liu YC, Tu CL. Psychometric properties of the Chinese version of the Swanson, Nolan, and Pelham, version IV scale - parent form. Int J Methods Psychiatr Res. 2008;17(1):35-44. doi: 10.1002/mpr.237. PMID: 18286459; PMCID: PMC6878250.
15. Fischer J, Krishnamurthy J, Hansen S, Reeves PT. Austere Foreign Body Injuries in Children and Adolescents: A Characterization of Penile, Rectal, and Vaginal Injuries Presenting to Emergency Departments in the United States From 2008 to 2017. Pediatr Emerg Care. 2021 Dec 1;37(12):e805-e811. doi: 10.1097/PEC.0000000000001803. PMID: 30870345.
16. Kellogg ND. Sexual behaviors in children: evaluation and management. Am Fam Physician. 2010 Nov 15;82(10):1233-8. PMID: 21121534.
17. Poli F, Meyer M, Mars RB, Hunnius S. Exploration in 4-year-old children is guided by learning progress and novelty. Child Dev. 2025 Jan-Feb;96(1):192-202. doi: 10.1111/cdev.14158. Epub 2024 Sep 2. PMID: 39223863; PMCID: PMC11693834.
18. Qin Y, Omar B, Musetti A. The addiction behavior of short-form video app TikTok: The information quality and system quality perspective. Front Psychol. 2022 Sep 6;13:932805. doi: 10.3389/fpsyg.2022.932805. PMID: 36148123; PMCID: PMC9486470.
19. Wang L, Shu X, Huang J, Yan W, Zhao D. Quality and Reliability of Adolescent Sexuality Education on Chinese Video Platforms: Sentiment-Topic Analysis and Cross-Sectional Study. JMIR Form Res. 2025 Sep 5;9:e77100. doi: 10.2196/77100. PMID: 40910701; PMCID: PMC12449667.
20. Griffiths S, Harris EA, Whitehead G, Angelopoulos F, Stone B, Grey W, Dennis S. Does TikTok contribute to eating disorders? A comparison of the TikTok algorithms belonging to individuals with eating disorders versus healthy controls. Body Image. 2024 Dec;51:101807. doi: 10.1016/j.bodyim.2024.101807. Epub 2024 Nov 5. Erratum in: Body Image. 2025 Mar;52:101834. doi: 10.1016/j.bodyim.2024.101834. PMID: 39504757.
21. Brand C, Fochesatto CF, Gaya AR, Schuch FB, López-Gil JF. Scrolling through adolescence: unveiling the relationship of the use of social networks and its addictive behavior with psychosocial health. Child Adolesc Psychiatry Ment Health. 2024 Aug 31;18(1):107. doi: 10.1186/s13034-024-00805-0. PMID: 39217325; PMCID: PMC11365153.
22. Lissak G. Adverse physiological and psychological effects of screen time on children and adolescents: Literature review and case study. Environ Res. 2018 Jul;164:149-157. doi: 10.1016/j.envres.2018.01.015. Epub 2018 Feb 27. PMID: 29499467.
23. Harness J, Getzen H. TikTok's Sick-Role Subculture and What to Do About It. J Am Acad Child Adolesc Psychiatry. 2022 Mar;61(3):351-353. doi: 10.1016/j.jaac.2021.09.312. Epub 2021 Sep 14. PMID: 34534625.
24. N Nagata JM, Shim JE, Balasubramanian P, Cheng CM, Al-Shoaibi AAA, Shao IY, Ganson KT, Testa A, Kiss O, He J, Baker FC. Prospective Associations Between Early Adolescent Problematic Screen Use, Mental Health, Sleep, and Substance Use. Am J Prev Med. 2026 Apr;70(4):108248. doi: 10.1016/j.amepre.2025.108248. Epub 2026 Feb 12. PMID: 41677479; PMCID: PMC12990317.
25. Fineberg NA, Demetrovics Z, Potenza MN, Mestre-Bach G, Ekhtiari H, Roman-Urrestarazu A, Achab S, Kattau T, Bowden-Jones H, Thomas SA, Babor TF, Kidron B, Stein DJ. Global action on problematic usage of the internet: announcing a Lancet Psychiatry Commission. Lancet Psychiatry. 2025 Jan;12(1):11-13. doi: 10.1016/S2215-0366(24)00323-7. Epub 2024 Oct 17. PMID: 39427681.
26. Selak Š, Horvat J, Žmavc M. Problematic Digital Technology Use Measures in Children Aged 0 to 6 Years: Scoping Review. JMIR Ment Health. 2025 Mar 18;12:e59869. doi: 10.2196/59869. PMID: 40100254; PMCID: PMC11962332.
